# ADHD Symptoms in Pathological and Problem Gamblers in Singapore

**DOI:** 10.3390/ijerph15071307

**Published:** 2018-06-22

**Authors:** Charles Mak, Kok Kah Tan, Song Guo

**Affiliations:** National Addictions Management Service, Institute of Mental Health, Buangkok Green Medical Park, 10 Buangkok View, Singapore 539747, Singapore; lawrence1_@hotmail.com (K.K.T.); song_guo@imh.com.sg (S.G.)

**Keywords:** attention-deficit/hyperactivity disorder, ADHD, PG, pathological gambling, pathological gamblers, problem gambling, problem gamblers, gambling addiction, impulsivity

## Abstract

*Background*: There is relatively little research examining the relationship between Attention-Deficit/Hyperactivity Disorder (ADHD) and gambling addiction. This study seeks to explore for ADHD symptoms in adult gambling addiction patients and to evaluate their gambling-related cognitions. *Materials and Methods*: A cross-sectional survey was conducted at National Addictions Management Service, Institute of Mental Health, in Singapore. Patients presenting for gambling treatment were screened for ADHD symptoms and assessed for severity of gambling-related cognitions. The primary objective was to observe the rate of patients screening positive for ADHD. *Results*: 20% of the sample screened positive for ADHD. These individuals also had lower levels of gambling-related cognitions. No significant correlation was noted between ADHD symptoms and gambling-related cognition scores. *Conclusions*: Positive screening results for ADHD occurred frequently in our sample of Pathological Gambling (PG) and Problem Gambling patients and these affected individuals also exhibited lower levels of gambling-related cognitions. This finding may suggest that the gambling behavior in patients with ADHD-PG comorbidity is driven by impulsivity rather than gambling-related cognitions, which has implication on treatment considerations. Further research with a larger sample size is indicated.

## 1. Introduction

The presence of Attention-Deficit/Hyperactivity Disorder (ADHD) may have an influence on the genesis, perpetuation and treatment of Pathological Gambling (PG) [1]. ADHD prevalence is well studied; with the National Comorbidity Survey Replication (NCS-R) suggesting a prevalence of 4.4% of the condition in adults, and various prevalence studies on ADHD in adult populations providing a pooled prevalence of 2.5% in a meta-analysis [2]. Comorbid substance addiction has been estimated to affect up to half of adults with ADHD [3]. Research into other addictive behaviors suggests an association between ADHD and gambling addiction [4]. Despite this, a tendency exists for adult ADHD to remain undiagnosed, in particular for patients with addiction problems [5].

Pathological Gambling and ADHD share a common feature of impulsivity. The Diagnostic and Statistical Manual for Mental Disorders, Fifth Edition (DSM-5), classifies Gambling Disorder as an addictive disorder. Historically, Pathological Gambling was classified as an impulse-control disorder in the Diagnostic and Statistical Manual for Mental Disorders, Fourth Edition (DSM-IV), and in ADHD, impulsivity symptoms contribute to the diagnostic criteria. Functional imaging studies in ADHD patients have demonstrated atypical fronto-striatal activation when tasks involving switching between response alternatives and inhibition are performed [6]. Patients with damage to the ventromedial frontal cortex are known to exhibit impulsive gambling behavior, in making risky choices on the Iowa gambling task, and increasing betting in presence of normal probability judgments [7,8,9]. Other studies have found impulsivity to be associated with both pathological and problem gambling, particularly in young adults [10,11,12,13,14].

The concept of an ‘Impulsive Gambler’ subtype is one that has been proposed by various researchers in this field. In the 1970s, Moran [15] discussed the concept of the ‘impulsive gambler’, with such gamblers having problems with strong urges to gamble, loss of control over their gambling habits, and serious social and economic dysfunction as a result of their gambling. Zimmerman et al. [16] sought a more objective basis for classification in 1985, utilizing factor analysis of Inventory of Gambling Behavior responses from gambling patients and non-gambling controls. Pathological gamblers with higher scores on items indicating high energy levels and risk-taking behaviors tended to describe themselves as energetic risk takers.

The ADHD-PG comorbidity may therefore be an overlooked but rational explanation for an apparent subtype of gambling patients with marked impulsivity features. This subgroup of patients may require assessment and treatment for impulsivity problems alongside conventional treatment.

There is a relative lack of research on the ADHD-PG comorbidity, in comparison to literature on the link between substance addiction and ADHD. To the best of our knowledge, there is no study done on an Asian population to explore this comorbidity. The few existing studies which have examined the ADHD-PG comorbidity have suggested such a correlation in both adolescents and adulthood [17,18,19,20]. A recent study by Grall-Bronnec et al. [5] has suggested a correlation between adult ADHD with higher severity of gambling problems.

In view of this information gap and the implications of the ADHD-PG comorbidity on the understanding and management of gambling addiction, our study team proposed a cross-sectional study to explore this comorbidity in Singaporean adults. The hypothesis was that an adult gambling treatment population in Singapore would screen positive for ADHD at a higher rate in comparison to the general adult population. The primary objective was to observe the rate of patients screening positive for ADHD in a population seeking treatment for gambling addiction. Secondary objectives were to observe the level of gambling-related cognitions in these patients, and to investigate for correlation between ADHD symptoms with gambling-related cognition scores.

## 2. Materials and Methods

### 2.1. Research Design

A cross-sectional single-center study was conducted at National Addictions Management Service (NAMS), Institute of Mental Health (IMH), in Singapore, to examine ADHD symptoms in patients with gambling problems.

All subjects gave their informed consent for inclusion before they participated in the study. The study was conducted in accordance with the Declaration of Helsinki, and the protocol was approved by the National Healthcare Group (NHG) Domain Specific Review Board (2013/00557).

### 2.2. Participant Criteria

Target study participants were new cases presenting with gambling addiction problems. Individuals aged between 21 to 65 years with a diagnosis of either Pathological Gambling or Problem Gambling were eligible for the study. Exclusion criteria included substance misuse problems, acute problems with mental health (i.e., severely depressed and/or psychotic), marked cognitive impairment, or if they were pregnant women or prisoners.

### 2.3. Data Collection

The data collection period was between 1 October 2013 and 31 March 2016. Following assessment by a clinician, consenting participants were subjected to a set of self-report survey instruments. Socio-demographic data was extracted from electronic patient records at IMH.

### 2.4. Adult ADHD Self-Report Scale (ASRS-v1.1)

The Adult ADHD Self-Report Scale (ASRS-v1.1) [21,22,23] is a self-report scale of adult ADHD. It was developed in conjunction with the World Health Organization (WHO) and the Workgroup on Adult ADHD.

Specifically, we utilized the ASRS-v1.1 Screener, which is a subset of the ASRS-v1.1 which utilizes 6 out of the 18 questions in the full scale. One study found that the ASRS-v1.1 Screener outperformed the ASRS-v1.1 in identifying ADHD in the general population [21]. The ASRS-v1.1 Screener is acknowledged as a screening tool with acceptable validity for identifying ADHD in addictions patients [23], with sensitivity of 87.5% and a negative predictive value of 95.7%. 

Based on the results of the ASRS-v1.1 Screener, participants were classified into two groups of ADHD status. A positive screening result for ADHD would result in a classification of ‘Likely ADHD’, while a negative screening result resulted in a classification of ‘Not likely ADHD’.

### 2.5. Gambling Attitudes and Beliefs Survey (GABS)

The GABS (Breen and Zuckerman, 1999) [24] is a 35-item self-rated questionnaire which measures gambling-related cognitions; such as cognitive bias, irrational beliefs, attitudes to gambling and gambling behaviors. The GABS is scored as the mean of all items, with a higher overall score indicating the presence of irrational pro-gambling attitudes and beliefs (mean GABS scores in non-problem gamblers = 76.07, mean GABS scores in problem gamblers = 92.11, mean GABS scores in pathological gamblers = 92.65) [5].

### 2.6. Diagnostic Criteria for Pathological Gambling

Patients were assessed with a 10-item checklist based on the 10 diagnostic criteria of the DSM-IV for pathological gambling, and from this, we were able to obtain 2 groups of gambling status: ‘Problem Gambling’ (4 or less criteria), and ‘Pathological Gambling’ (5 or more criteria).

### 2.7. Statistical Analyses

All data were analyzed using the computer based statistical software package SPSS (IBM SPSS Statistics 22). Descriptive statistics were calculated for the sample population and for subgroups of ADHD and gambling status. Assumptions of random sampling and normality of distributions in the population were examined to ensure that the underlying statistical assumptions were upheld. Tests of significance were set at *p* < 0.05.

Pearson’s correlation coefficients were computed to examine the associations between ADHD symptoms and gambling-related cognitions.

## 3. Results

### 3.1. Socio-Demographics

Table 1 provides an overview of the demographic characteristics of the sample. The sample comprised 64 males (98.5%) and 1 female (1.5%). Fifty-six participants were Chinese (86.2%), 4 participants were Malays (6.2%) and 5 participants were Indians (7.7%). Seventeen participants were single (26.2%), 36 were married (55.4%) and 12 were divorced or separated. The majority of the participants had no children (70.8%). The mean age of the sample was 36 years old. Two participants had primary school education (3.1%), 24 participants had secondary education (36.9%), 26 participants had tertiary education (40%) and 13 had either graduate or post-graduate education (20%). Fifty-two of the participants were either in full-time or part-time employment (80%), and 13 participants were unemployed (20%). 

### 3.2. Gambling and Clinical Characteristics

In Table 2, 13 (20%) of 65 participants were found to be in the ‘Likely ADHD’ group. All of these participants also met diagnostic criteria for Pathological Gambling. The chi-square test for independence was conducted to explore a relationship between ADHD and gambling status. No statistically significant association was found between ADHD and gambling status on the condition of χ(1) = 0.786 and *p* = 0.375.

Table 3 refers to the mean scores for Pathological Gambling criteria according to ADHD status. Patients with ‘Likely ADHD’ status had a lower mean of Pathological Gambling criteria (7.1) compared to patients with the ‘Not likely ADHD’ status (7.3). The independent-samples *t*-test was conducted to compare the means of Pathological Gambling criteria according to ADHD status. Patients with the ‘Likely ADHD’ status did not have statistically significantly higher Pathological Gambling criteria (7.31 ± 1.25) compared to patients with the ‘Not likely ADHD’ status (7.1 ± 1.76) for *t*(63) = −0.419 and *p* = 0.677.

Table 4 refers to the means and standard deviations of GABS scores. The mean GABS score for the ‘Likely ADHD’ group was 89 (SD = 14), while the mean GABS score for the ‘Unlikely ADHD’ group was 84 (SD = 16). The independent-samples *t*-test was conducted to compare the means of GABS scores according to ADHD status. Patients with the ‘Likely ADHD’ status did not have statistically significantly higher GABS score (83.92 ± 16.29) compared to patients with the ‘Not likely ADHD’ status (89.12 ± 13.40) for *t*(63) = 1.197 and *p* = 0.236.

### 3.3. Associations between ADHD Symptoms and Gambling-Related Cognitions 

Table 5 refers to Pearson’s correlation coefficients. No correlations were found between ADHD symptoms and gambling-related cognitions.

## 4. Discussion

### 4.1. Primary Objective

In our sample of adult patients seeking treatment for gambling addiction in Singapore, 20% screened positive for ADHD when assessed with the ASRS-v1.1 Screener. In comparison, the NCS-R provided a prevalence rate of 4.4% of adult ADHD, while in a 2012 study on a primary care population, 15% of the sample screened positive for ADHD on the ASRS-v1.1 Screener [25]. This supports our hypothesis that the adult gambling-treatment population in Singapore would screen positive for ADHD at a higher rate in comparison to the adult general population.

### 4.2. Gambling-Related Cognitions in Relation to ADHD

The sample as a whole attained high mean scores on the GABS. However, the ‘Likely ADHD’ group attained a lower mean score of 84 on the GABS, compared to the mean score of 89 for the ‘Not Likely ADHD’ group.

There is limited available research on the frequency of dysfunctional gambling-related cognitions in relation to ADHD, although a similar study found that patients who exhibited the ADHD-PG comorbidity featured higher levels of gambling-related cognitions [5]. A more recent longitudinal case-control study also found associations of the presence of ADHD with elevated gambling-related cognitions [26]. Therefore, our finding is not in keeping with limited available research. Our study also did not identify significant correlations between ADHD symptoms and gambling-related cognitions, which may be related to the relatively low number of participants recruited. 

It would be interesting if this finding is replicated in further studies. A possible explanation for this finding could be that impulsivity drives gambling activity in patients with ADHD symptoms. In contrast, for patients without ADHD comorbidity, gambling-related cognitions may be primarily responsible for gambling activity. This would have implications on treatment. Pharmacological treatment for ADHD and psychological interventions to address problems of impulsivity, alongside conventional gambling treatment options, may contribute to obtaining a better treatment outcome in patients with the ADHD-PG comorbidity.

### 4.3. The Profile of the Singaporean Pathological Gambler

The sample primarily consisted of participants of Chinese origin (86.2%), males (98.5%) and a mean age of 36 years. 96.9% of the sample possessed at least secondary school education and 80% were employed. Gambling has always been an integral part of Chinese culture [27]. Unique to the Chinese culture is that gambling is seemingly intertwined with the Chinese belief in luck, which has been the subject of research and led to the construction of the Chinese good luck scale [28].

The predominant characteristics of being middle-aged, higher education status and active employment may be indicators that Singaporean pathological gamblers are more financially successful compared to pathological gamblers in other parts of the world. In a study on another population made up of predominantly Chinese participants in Hong Kong, the majority of pathological gamblers were found to have less than 9 years of education [29]. A qualitative study on youth gambling conducted in Canada found lower socio-economic status to be a central theme among youth gamblers [30].

This unique profile of the Singaporean pathological gambler may be attributable to cultural and social acceptance of gambling as a viable pastime among well-established and financially successful individuals, and the need for stable financial resources to engage in gambling activities, for example, to incur the cost of an entry levy to enter a casino in Singapore.

In Singapore, mobile phones and smartphones are the most commonly utilized platforms for gambling online [31] and smartphones often provide an immersive virtual reality environment for users, resulting in Internet Gaming Disorder [32]. It is important to note that both Internet Gaming Disorder [33] and Internet Addiction [34] are associated with ADHD. There is a possibility that these conditions are mediating factors between Pathological Gambling and ADHD. Further research is required to study the mediating effect.

### 4.4. Other Study Limitations

The ASRS-v1.1 Screener is a screening tool and does not diagnose ADHD. A consideration for future studies would be to use instruments for purposes of diagnostic assessment, such as the physician-administered Adult ADHD Clinical Diagnostic Scale (ACDS) [35] or the semi-structured Diagnostic Interview for Adult ADHD (DIVA) [36]. In addition, participants who score highly on the ASRS-v1.1 Screener may be suffering from separate illness, conferring increased levels of impulsivity. We attempted to minimize this possibility by excluding patients with acutely unstable mental status.

This study was conducted in only one setting; at the outpatient clinic at NAMS. Patients seen here may be suffering from more severe illness than in the general gambling addiction population, and stigma may have influenced non-attendance by certain groups.

Patients unable to understand English were not explicitly excluded from the study, but only the English versions of selected self-report tools were used. This has led to a problem with sampling bias, with participants of a lower education level and lack of exposure to the English language potentially being declined recruitment into the study.

## 5. Conclusions

This study found a high rate of positive screening results for ADHD in patients presenting for gambling addiction treatment at an addiction treatment center in Singapore. Patients with the ‘Likely ADHD’ status tended to have lower levels of gambling-related cognitions, which may indicate that the gambling behavior in patients with ADHD-PG comorbidity is driven by impulsivity, rather than distorted gambling-related cognitions.

These findings may have implications on conventional approaches to the management of pathological gambling. Routine screening for the presence of ADHD and providing treatment options to address the impulsivity aspects of the condition may add value to the overall treatment outcome.

This study did not find significant correlation between ADHD symptoms and gambling-related cognitions. Despite this and its limitations, this study has provided insight into an area where an information gap exists. Further studies with a higher number of participants will be of much interest.

## Figures and Tables

**Table 1:** Socio-demographics of sample (n = 65).

Variable	Category	Mean/N	S.D./Proportion
Age		36.3	11.1
Gender	Male	64	98.5
	Female	1	1.5
Ethnic group	Chinese	56	86.2
	Malay	4	6.2
	Indian	5	7.7
Marital status	Married	36	55.4
	Divorced	12	18.5
	Single	17	26.1
Number of children	4	1	1.5
	3	3	4.6
	2	11	16.9
	1	4	6.2
	0	46	70.8
Highest educational status attained	Graduate or Post-Graduate	13	20
	Tertiary	26	40
	Secondary	24	36.9
	Primary	2	3.1
Employment status	Employed	52	80
	Unemployed	13	20

**Table 2:** ADHD and gambling status.

	Proportion
‘Likely ADHD’	20
‘Not likely ADHD’	80
Pathological Gambling	95
Problem Gambling	5

**Table 3:** Pathological Gambling criteria according to ADHD status.

	Mean	SD
‘Likely ADHD’	7.1	1.7
‘Not likely ADHD’	7.3	1.3

**Table 4:** Means and standard deviations of GABS scores.

	Mean	SD
‘Likely ADHD’	84	14
‘Not likely ADHD’	89	16

**Table 5:** Associations between ADHD symptoms and gambling-related cognitions.

	GABS	ASRS
GABS		
Pearson Correlation	1	−0.176
Sig. (2-tailed)		0.169
N	65	65
ASRS		
Pearson Correlation	−0.176	1
Sig. (2-tailed)	0.160	
N	65	65
